# Effectiveness and safety of Baduanjin exercise (BDJE) on heart failure with preserved left ventricular ejection fraction (HFpEF): A protocol for systematic review and meta-analysis

**DOI:** 10.1097/MD.0000000000022994

**Published:** 2020-11-13

**Authors:** Mingtai Chen, Lijun Ou, Yingnan Chen, Ling Men, Xiaoling Zhong, Shudong Yang, Jienan Luan

**Affiliations:** a Department of Cardiovascular Disease; b Nephrology Department; c Reproductive Health Department, Shenzhen Traditional Chinese Medicine Hospital, Shenzhen, China.

**Keywords:** Baduanjin, heart failure with preserved left ventricular ejection fraction, meta-analysis, protocol, randomized trial

## Abstract

**Background:**

Nearly half of the heart failure (HF) patients have been classified as HF with preserved left ventricular ejection fraction (HFpEF) and the prevalence has been increasing over time. The subject of this study is to assess the clinical *effectiveness* and safety of Baduanjin exercise (BDJE), as a kind of traditional Chinese exercises, for HFpEF patients.

**Methods:**

A systematic literature search for articles up to September 2020 will be performed in following electronic databases: PubMed, Embase, the Cochrane Library, China National Knowledge Infrastructure (CNKI), Chinese Scientific Journals Database (VIP) Database, Chinese Biomedical Database (CBM), Chinese Biomedical Literature Service System (SinoMed) and Wanfang Database. Inclusion criteria are randomized controlled trials of BDJE applied on HFpEF patients. The primary outcome measures will be exercise capacity (cardiopulmonary exercise test or 6-minute walking test) and quality of life. The secondary outcomes will be as the following: blood pressure, heart rate, echocardiography, endothelial function, arterial stiffness and hypersensitive C-reactive protein and N-Terminal pro-B-type natriuretic peptide. The safety outcome measures will be adverse events, liver and kidney function. RevMan 5.3 software will be used for data synthesis, sensitivity analysis, subgroup analysis and risk of bias assessment. A funnel plot will be developed to evaluate reporting bias. Stata 12.0 will be used for meta-regression and Egger tests. We will use the Grading of Recommendations Assessment, Development and Evaluation (GRADE) system to assess the quality of evidence.

**Conclusion:**

The study will give an explicit evidence to evaluate the effectiveness and safety of BDJE for HFpEF patients.

**Ethics and dissemination:**

This systematic review does not require ethics approval and will be submitted to a peer-reviewed journal.

**Trial registration number:**

PROSPERO CRD42020200324.

## 1 Introduction

Heart failure (HF), as a kind of complicated clinical syndrome, has been recognized as the terminal stage of various cardiovascular diseases. Because of the high morbidity, mortality and the heavy medical burden of HF, it has been a global health issue.^[1]^ Heart failure with preserved left ventricular (LV) ejection fraction (HFpEF), as an important classification of HF, has been characterize by the presence of signs and symptoms of heart failure without evidence of reduced LV ejection fraction. Nearly half of the HF patients have been classified as HFpEF and the prevalence has been increasing over time and HFpEF has been estimated to be the dominant type of HF in future.^[2,3]^ The pathological mechanisms in HFpEF patients have been highly heterogeneous, while various factors (such as left ventricular diastolic dysfunction, hypertension, atrial fibrillation, pulmonary hypertension, diabetes, aging, etc) contributed to the pathological process.^[4–7]^ The complicated pathological mechanisms of HFpEF have made developing therapeutic strategies for HFpEF more difficult.

Compared with the established and curative therapeutic strategies for heart failure with reduced ejection fraction (HFrEF), there have been not enough effective therapeutic strategies for HFpEF. In addition, conventional medical treatments for HFrEF have been proven not significantly beneficial for HFpEF patients.^[8]^ Therefore, not only more drug clinical trials are urgent, but also more diverse and comprehensive therapeutic strategies are needed to be explored. Exercise training, as a novel therapeutic approach of cardiac rehabilitation (CR) category, has been proven beneficial to HFpEF. Because exercise training has the advantages of safety and low cost, it has become more and more important in the field of HF. A meta-analysis showed that exercise training could significantly improve diastolic function in HFpEF patients.^[9]^ Furthermore, it was reported that exercise training could improve exercise capacity among HFpEF patients through the mechanisms of improving microvascular and skeletal muscle function.^[10,11]^ Moreover, exercise training could either improve endothelial function or neurohumoral regulation in HFpEF patients.^[12,13]^ As far as concerned, most of the HFpEF patients have been elderly and had exercise intolerance, therefore simple and easy learning exercise training is appropriate for them.

In Asia, traditional Chinese exercises including Baduanjin exercise (BDJE), Qigong and Tai Chi has been popular among elderly patients who are intolerant of moderate and high intense physical activities.^[14]^ Compared with Qigong and Tai Chi, BDJE is more convenient to conduct and easier to learn. It has been known that BDJE is composed of eight set of actions including support heaven with both hands, dragon sprays water with force, big bird spreads its wings, lift window to look at the moon on the left, descend to earth with force, beautiful maiden twists her waist to the right, extend shoulders to bring hands together, and dragon claws to the left.^[15]^ It has been proven that BDJE could reduce oxygen consumption of myocardia by relieving the cardiac burden and increasing utilization rate of oxygen in blood circulation.^[16]^ In addition, clinical trials indicated that BDJE could regulate blood glucose metabolism, immune function, inflammatory response, which were critical risk factors contributing to the pathological process of HFpEF, by regulating expression of associated lncRNA, mRNA and circRNA, involving IL-17 and TNF signaling pathways.^[17]^ Furthermore, it has been reported that BDJE could also protect endothelial function and blood vessel elasticity by suppressing oxidative stress, which could relieve myocardial injury in HFpEF.^[15,18–20]^ Several clinical trials also indicated that BDJE could improve fatigue, quality of life (QoL) and reverse adverse LV remodeling in HF patients.^[21,22]^ Moreover, BDJE was beneficial for systolic and diastolic blood pressure, and resting heart rate.^[23]^

Although many clinical studies indicated the benefits of BDJE to HFpEF patients, there were also some studies reported BDJE could not significantly improve blood pressure, heart rate and QoL.^[24]^ Besides the effectiveness of BDJE, the safety of BDJE for HFpEF patients is another crucial and concerned issue, which was reported rarely.

However, there has been no systematic review evaluating the effectiveness and safety of BDJE on HFpEF patients yet. In view of the shortcomings of previous studies and the insufficient evidence regarding the widespread application of BDJE on HFpEF patients, an objective and systematic evaluation is needed. Therefore, we will conduct a systematic review and meta-analysis aiming to summarize the effectiveness and safety of BDJE on HFpEF patients.

## 2 Methods and analysis

### 2.1 Registration

The study protocol has been registered in the international prospective register of systematic review (PROSPERO). The trial registration number of PROSPERO is CRD42020200324. The procedure of this protocol will be conducted according to the Preferred Reporting Item for Systematic Review and Meta-analysis Protocols (PRISMA-P) guidelines.^[25]^

### 2.2 Eligibility criteria

#### 2.2.1 Type of study

##### 2.2.1.1 Inclusion

We will include all the RCTs that investigated the effectiveness and safety of BDJE combined with conventional pharmacotherapy for the treatment of HFpEF patients.

##### 2.2.1.2 Exclusion

The studies will be excluded if it is not an RCT (namely, observational cohort and case–control studies, case reports, experimental studies and reviews).

#### 2.2.2 Participants

##### 2.2.2.1 Inclusion

The study will include adult (18–85 years) HFpEF patients regardless of sex, ethnicity, education or economic status and whether or not they were out- or in-patients. The diagnostic criteria of HFpEF should be confirmed according to one of the past or current definitions: the Heart Failure Association (HFA) of the European Society of Cardiology (ESC)^[26]^ or the American College of Cardiology Foundation (ACC) / American Heart Association (AHA) guideline for the management of heart failure.^[27]^

##### 2.2.2.2 Exclusion

Patients with severe respiratory disease, acute infectious disease, severe heart disease, severe liver disease or tumors will be excluded.

#### 2.2.3 Interventions

##### 2.2.3.1 Inclusion

Eligible interventions will be those involving a combination of BDJE and conventional pharmacotherapy. The same conventional pharmacotherapy must be used in the control group.

##### 2.2.3.2 Exclusion

Trials that include other co-interventions such as acupuncture, cupping, moxibustion, massage, yoga, qigong, Tai Chi, or aromatherapy will be excluded.

#### 2.2.4 Outcome

##### 2.2.4.1 Inclusion

The primary outcome measures will include the followings: exercise capacity, which is assessed by cardiopulmonary exercise test (CPET) or 6-minute walking test (6MWT), and QoL. The secondary outcomes are the followings: blood pressure, heart rate, echocardiography (ventricular function and left ventricular diastolic function), endothelial function, arterial stiffness and hypersensitive C-reactive protein (hsCRP) and N-Terminal pro–B-type natriuretic peptide (NT-proBNP). The safety outcomes will include the followings: adverse events (such as digestive symptoms, headache, dizziness, skin rash, etc), liver or kidney toxicity measured by serum markers.

##### 2.2.4.2 Exclusion

The outcome measures not requested in this study will be excluded.

### 2.3 Search strategy

The following electronic bibliographic databases will be searched from inception to September 2020: PubMed, Embase, the Cochrane Library, China National Knowledge Infrastructure (CNKI), Chinese Scientific Journals Database (VIP) Database, Chinese Biomedical Database (CBM), Chinese Biomedical Literature Service System (SinoMed) and Wanfang Database. A manual search of key journals and of the reference lists of reviews captured by the initial searches will also be performed. There will be no limits on the language of publication. Only clinical trials will be included and searched. The following sources will also be searched to identify clinical trials that are in progress or completed: Clinical Trials.gov and WHO clinical trials registry. Any additional relevant studies will also be retrieved from the reference lists of systematic reviews and included studies. If possible, we will map search terms to controlled vocabulary. In addition, the search strategy for selecting the fields of title, abstract or keyword will differ depending on the characteristics of the databases. Search terms will be grouped into three blocks (see Table 1).

**Table 1:** Search items.

Search Block	Search items
Participants	Cardiac Failure OR Heart Decompensation OR Heart Failure OR Right-Sided Heart Failure OR Myocardial Failure OR Congestive Heart Failure OR Left Sided Heart Failure OR Preserved Ejection Fraction OR Preserved Ejection Fraction Heart Failure OR Heart Failure, Preserved Ejection Fraction OR Diastolic heart failure OR Heart failure with preserved left ventricular ejection fraction OR HFpEF
Intervention	Baduanjin exercise OR Baduanjin OR BDJ OR BDJE OR Qigong OR eight section brocades OR regimen OR Chinese regimen OR Chinese ancient regimen OR rehabilitation exercise OR Medicine, Chinese Traditional OR Traditional Chinese Medicine OR Chung I Hsueh OR Hsueh, Chung I OR Traditional Medicine, Chinese OR Zhong Yi Xue OR Chinese Traditional Medicine OR Chinese Medicine, Traditional
Study design	Randomized controlled trial OR controlled clinical trial OR randomized OR placebo OR drug therapy OR randomly OR trial OR groups

### 2.4 Study selection and data extraction

**Figure 1:**
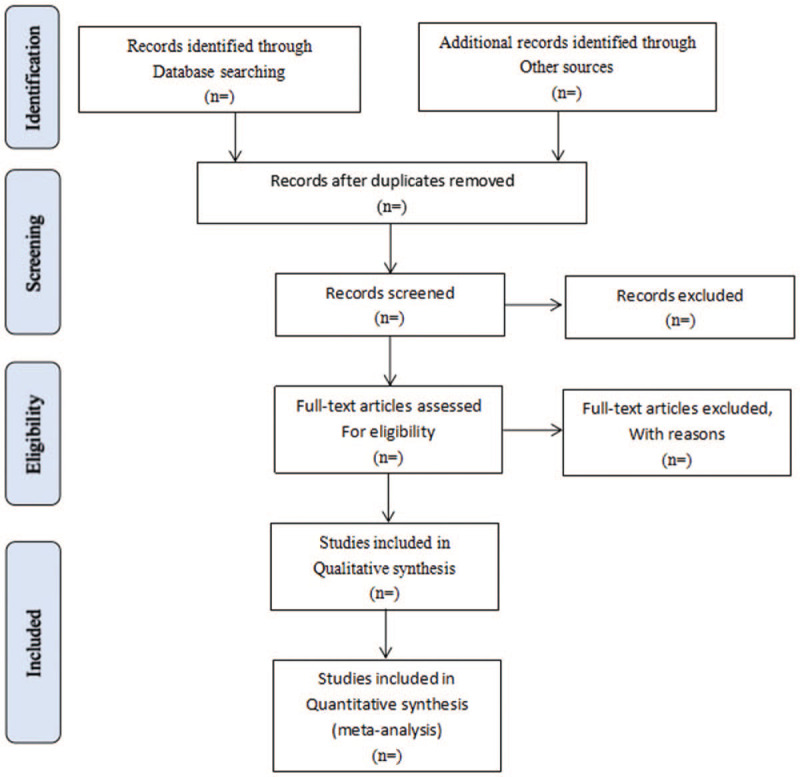
Flow diagram of study selection process. PubMed, Embase, the Cochrane Library, CNKI, VIP Database, CBM, SinoMed and Wanfang Database.

Literature retrieved citations will be managed by EndNote X7 software. Two authors (MC and YC) will independently screen the titles and abstracts of all the studies retrieved in the above electronic databases to identify potentially eligible studies. Articles that are duplicated or have not met the eligibility criteria, interventions and outcomes in this study will be excluded. After filtering the final eligible articles, the data from the included articles will be extracted independently by2 authors (MC and LM). Disagreements will be resolved by discussion or arbitrated by a third author if needed. The following categories of data will be extracted: first author, publication year, diagnose information, age, sex, trial characteristics, interventions and controls, participants, study methodology, outcomes, and adverse events (see Fig. 1).

### 2.5 Risk of bias assessment

The methodological quality of the eligible studies will be evaluated according to the Cochrane Collaboration's tool for assessing risk of bias. The assessment details include: sequence generation, allocation concealment, blinding of participants and personnel, blinding of outcome assessors, incomplete outcome data, selective reporting and other sources of bias. Each domain will be assessed as “low risk”, “high risk” or “unclear risk” according to the description details of eligible studies.

### 2.6 Data synthesis and statistical analysis

Statistical analyses will be conducted with RevMan 5.3 software provided by Cochrane Collaboration. The overall effect sizes will be determined as the mean difference (MD) for continuous outcomes, the odds ratio (OR) for dichotomous outcomes with their 95% credible intervals (CIs). The Q and I^2^ test statistics will be calculated to determine the amount of heterogeneity. For the Q statistic, *P* < .05 will be considered to indicate significant differences. For the I^2^ statistic, I^2^ < 25% indicates no significant heterogeneity, I^2^ = 25%–50% is considered moderate heterogeneity and I^2^ > 50% indicates strong heterogeneity. We will use fixed effects models if there is no heterogeneity among studies, and random effects models if there is heterogeneity.

### 2.7 Sensitivity analysis, subgroup analysis, and meta-regression

If the heterogeneity or inconsistency among the studies is detected, a sensitivity analysis or subgroup analysis or meta-regression (conducted by Stata 12.0) analysis will be performed. Subgroup analysis will be conducted to explore potential sources of heterogeneity according to the characteristics of studies, including sample size, New York Heart Association classification of cardiac function, severity of exercise intolerance, treatment frequency, treatment duration and other relevant parameters. If data extraction is insufficient, we will create a qualitative synthesis.

### 2.8 Publication bias

A funnel plot will be developed to evaluate reporting bias of the included studies. We will use Egger tests (conducted by Stata 12.0) to assess funnel plot symmetry and will interpret values of *P* < .1 as statistically significant.

### 2.9 Quality of evidence

We will also assess the quality of evidence for the main outcomes with the Grading of Recommendations Assessment, Development and Evaluation (GRADE) approach. Five items will be investigated, including limitations in study design, inconsistency, inaccuracies, indirectness and publication bias.

### 2.10 Patient and public Involvement

The patients and/or public will not be involved because this study uses secondary sources for analysis.

## 3 Discussion

We plan to conduct a systematic and meta-analysis to evaluate the effectiveness and safety of BDJE for HFpEF patients. However, there might be some limitations because this is a retrospective systematic and meta-analysis. Firstly, some unpublished studies will not be included because they might introduce some unexpected bias. In addition, some grey literature might be difficult to retrieve, leading to a selection bias in the literature. Moreover, the diagnosis criterion of HFpEF has been updated continuously so that there might be heterogeneity in included patients of different trials. Nevertheless, we hope that the results of this study will be able to propose the clinical recommendation for HFpEF patients in BDJE clinical practice and provide more reliable evidence for BDJE.

## Author contributions

**Conceptualization:** Lijun Ou, Xiaoling Zhong, Shudong Yang, Jienan Luan

**Data curation:** Mingtai Chen

**Formal analysis:** Mingtai Chen

**Funding acquisition:** Jienan Luan

**Investigation:** Lijun Ou

**Methodology:** Mingtai Chen

**Project administration:** Lijun Ou

**Resources:** Lijun Ou, Yingnan Chen

**Software:** Lijun Ou, Yingnan Chen

**Supervision:** Jienan Luan

**Validation:** Yingnan Chen

**Visualization:** Yingnan Chen, Ling Men

**Writing – original draft:** Mingtai Chen, Ling Men

**Writing – review & editing:** Xiaoling Zhong, Shudong Yang, Jienan Luan
